# (Acetonitrile){2-[bis­(pyridin-2-ylmethyl-κ^2^ *N*)amino-κ*N*]-*N*-(2,6-dimethyl­phen­yl)acetamide-κ*O*}(perchlorato-κ*O*)zinc (acetonitrile){2-[bis­(pyridin-2-ylmethyl-κ^2^ *N*)amino-κ*N*]-*N*-(2,6-dimethyl­phen­yl)acetamide-κ*O*}zinc tris­(perchlorate)

**DOI:** 10.1107/S1600536813001396

**Published:** 2013-01-19

**Authors:** Ove Alexander Høgmoen Åstrand, Carl Henrik Görbitz, Kenneth Aase Kristoffersen, Pål Rongved

**Affiliations:** a School of Pharmacy, University of Oslo, PO Box 1068 Blindern, N-0316 Oslo, Norway; b Department of Chemistry, University of Oslo, PO Box 1033 Blindern, N-0315 Oslo, Norway

## Abstract

In the title salt, [Zn(C_22_H_24_N_4_O)(CH_3_CN)][Zn(ClO_4_)(C_22_H_24_N_4_O)(CH_3_CN)](ClO_4_)_3_, two differently coordinated zinc cations occur. In the first complex, the metal ion is coordinated by the *N*,*N*′,*N*′′,*O*-tetra­dentate acetamide ligand and an acetonitrile N atom, generating an approximate trigonal–bipyramidal coordination geometry, with the O atom in an equatorial site and the acetonitrile N atom in an axial site. In the second complex ion, a perchlorate ion is also bonded to the zinc ion, generating a distorted *trans*-ZnO_2_N_4_ octa­hedron. Of the uncoordinating perchlorate ions, one lies on a crystallographic twofold axis and one lies close to a twofold axis and has a site occupancy of 0.5. N—H⋯O and N—H⋯(O,O) hydrogen bonds are observed in the crystal. Disordered solvent mol­ecules occupy about 11% of the unit-cell volume; their contribution to the scattering was removed with the SQUEEZE routine of the *PLATON* program [Spek (2009 ▶). *Acta Cryst.* D**65**, 148–155.].

## Related literature

For related structures found in the Cambridge Structural Database (Version 5.33 of November 2011; Allen, 2002 ▶), see: Xu *et al.* (2010*a* ▶,*b* ▶); Patten *et al.* (2008 ▶); Marlin *et al.* (2006 ▶). For biochemical background, see: Makhov *et al.* (2008 ▶); Xu *et al.* (2010*a* ▶).
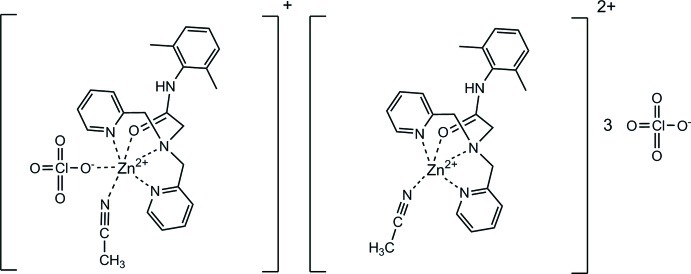


## Experimental

### 

#### Crystal data

[Zn(C_22_H_24_N_4_O)(C_2_H_3_N)][Zn(ClO_4_)(C_22_H_24_N_4_O)(C_2_H_3_N)](ClO_4_)_3_
*M* *_r_* = 1331.59
Monoclinic, 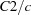

*a* = 41.253 (8) Å
*b* = 15.057 (3) Å
*c* = 20.809 (4) Å
β = 106.106 (2)°
*V* = 12418 (4) Å^3^
*Z* = 8
Mo *K*α radiation
μ = 1.03 mm^−1^
*T* = 105 K
0.91 × 0.29 × 0.22 mm

#### Data collection

Bruker APEXII CCD diffractometer
Absorption correction: multi-scan (*SADABS*; Bruker, 2007 ▶) *T*_min_ = 0.531, *T*_max_ = 0.798
43906 measured reflections
10987 independent reflections
8503 reflections with *I* > 2σ(*I*)
*R*_int_ = 0.051

#### Refinement

*R*[*F* ^2^ > 2σ(*F* ^2^)] = 0.045
*wR*(*F* ^2^) = 0.124
*S* = 1.07
10987 reflections
782 parameters
8 restraints
H-atom parameters constrained
Δρ_max_ = 0.89 e Å^−3^
Δρ_min_ = −0.66 e Å^−3^

### 

Data collection: *APEX2* (Bruker, 2007 ▶); cell refinement: *SAINT-Plus* (Bruker, 2007 ▶); data reduction: *SAINT-Plus*; program(s) used to solve structure: *SHELXTL* (Sheldrick, 2008 ▶); program(s) used to refine structure: *SHELXTL*; molecular graphics: *SHELXTL* and *Mercury* (Macrae *et al.*, 2008 ▶); software used to prepare material for publication: *SHELXTL*.

## Supplementary Material

Crystal structure: contains datablock(s) I, global. DOI: 10.1107/S1600536813001396/hb6972sup1.cif

Structure factors: contains datablock(s) I. DOI: 10.1107/S1600536813001396/hb6972Isup2.hkl

Additional supplementary materials:  crystallographic information; 3D view; checkCIF report

## Figures and Tables

**Table 1:** Selected bond lengths (Å)

Zn1*A*—N4*A*	2.058 (3)
Zn1*A*—N3*A*	2.059 (3)
Zn1*A*—N5*A*	2.060 (3)
Zn1*A*—O1*A*	2.087 (2)
Zn1*A*—N2*A*	2.236 (2)
Zn1*A*—O1*D*	2.310 (2)
Zn1*B*—N3*B*	2.020 (3)
Zn1*B*—O1*B*	2.025 (2)
Zn1*B*—N4*B*	2.040 (3)
Zn1*B*—N5*B*	2.043 (3)
Zn1*B*—N2*B*	2.240 (2)

**Table 2:** Hydrogen-bond geometry (Å, °)

*D*—H⋯*A*	*D*—H	H⋯*A*	*D*⋯*A*	*D*—H⋯*A*
N1*A*—H1*A*⋯O3*D* ^i^	0.88	2.00	2.868 (3)	171
N1*B*—H1*B*⋯O4*E* ^ii^	0.88	2.15	2.979 (3)	157
N1*B*—H1*B*⋯O3*E* ^ii^	0.88	2.41	3.138 (4)	141

Symmetry codes: (i) 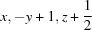; (ii) 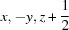.

**Fig. 1.**
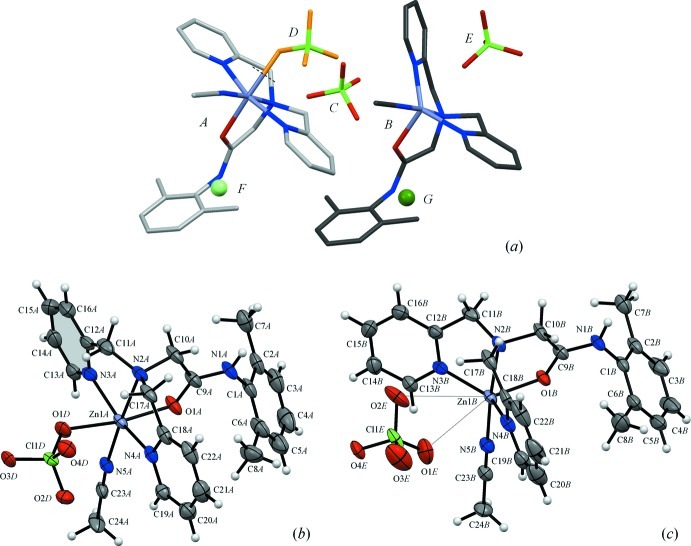
(a) The asymmetric unit with H atoms omitted for clarity. C atoms in molecule A (including the associated acetonitrile ligand) are shown in light grey colour, as opposed to C atoms in molecule B which are dark grey. O atoms in perchlorate ion D, coordinating to molecule A, appear in orange, while O atoms in perchlorate ion E close to B are dark red. Perchlorate ions F and G are depicted here as light green and green spheres, respectively, to reduce overlap with A and B. (b) Complex A with perchlorate D. One pyridine ring has been shaded. (c) Complex B with perchlorate E. The indicated distances are Zn1B···O1E = 3.077 (3) Å and Zn1B···O2E = 3.827 (3) Å, which may be compared to 2.310 (3) Å for O1D-Zn1A. Displacement ellipsoids in (b) and (c) are shown at the 50% probability level with H atoms as spheres of arbitrary size.

**Fig. 2.**
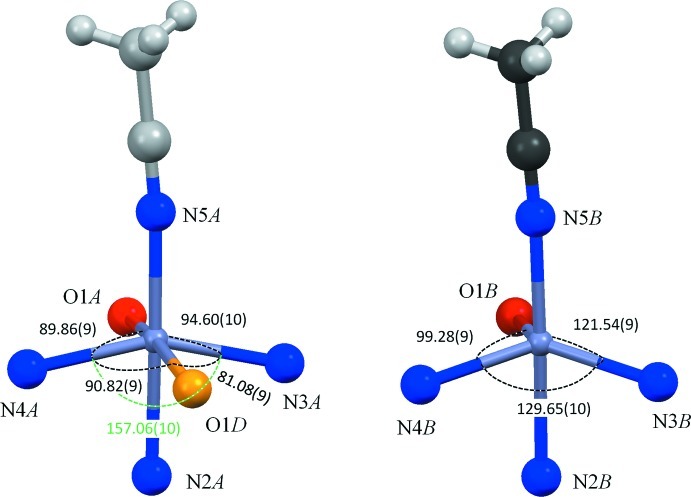
Octahedral geometry at Zn1A (left) and trigonal bipyramidal geometry at Zn1B (right). In-plane bond angles (°) have been indicated. N3—Zn1—N4 undergoes an opening of 27.4° from B to A to accommodate the extra perchlorate ligand O1D. In both complexes the N5—Zn1—N3/N4/O1 angles are slightly larger than the N2—Zn1—N3/N4/O1 angles, with ranges 96.0 - 102.8° and 78.7 - 80.3°, respectively.

**Fig. 3.**
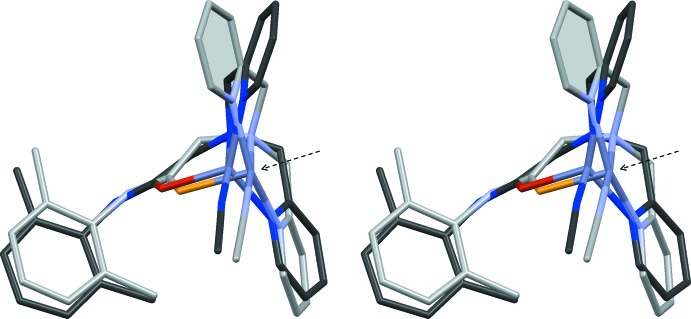
Stereo view of a molecular overlap between complex A and complex B. Colour coding as in Fig. 1(a), except that for molecule A N and O atoms are shown in lighter colours. The dashed arrow indicates the position of perchlorate D coordinating to A, which mainly serves to push the shaded ring system [see Fig. 1(b)] to the left.

**Fig. 4.**
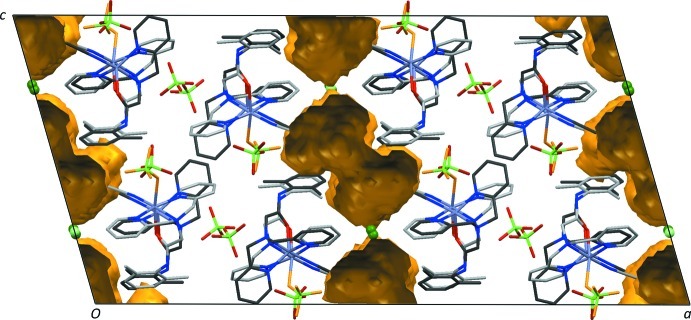
Molecular packing viewed along the b axis. Colour coding as in Fig. 1(a). The 'Display Voids' tool in Mercury (Macrae et al., 2008), with a 1.2 Å probe radius and 0.4 Å grid spacing, has been used to highlight regions of disordered solvent accounting for about 11% of the unit cell volume.

## supplementary crystallographic information

### Comment

The title compound (I) was prepared as part of a series of zinc-binding ligands to be tested biologically. Zinc chelation may induce apoptosis in prostate cancer cells (Makhov *et al.*, 2008), and may be used as Zn^2+^-selective, cell-permeable, and ratiometric fluorescent sensors (Xu *et al.*, 2010*a*) for biological mapping of disease. The title compound was synthesized from 2,6-dimethyl aniline, chloroacetyl chloride and *N*,*N*'-dipicolylamine in a two-step procedure as a part of a larger work that will be published in due time.

The asymmetric unit is shown in Fig. 1 (*a*). Two independent 2-[bis(pyridin-2-ylmethyl)amino]-*N*-(2,6-dimethylphenyl) acetamides *A* and *B* act as tetradentate ligands for Zn^2+^ ions. Cocrystallized acetonitrile solvent molecules serve as additional ligands. There are five different perchlorate anions *C*, *D*, *E*, *F* and *G*, the latter two being located on (*F*) or very close to (*G*) a twofold axis. Perchlorate *D* serves as ligand number six for Zn1*A*, Fig. 1 (*b*), while perchlorate E is only loosely connected to Zn1B, Fig. 1(*c*). This means that Zn1*A* is octahedrally coordinated, while Zn1*B* has distorted trigonal bipyramidal geometry, Fig. 2. The effect on the overall molecular geometries is evident from the overlay in Fig. 3, which yields a RMS deviation of 0.76 Å. Torsion angle deviations between the two complexes are usually in the range 0 – 20°, but reaches 68.5° for C17—N2—C11—C12 [*A*: -85.1 (3)°; *B*: -153.6 (3)°]. The unit cell and crystal packing arrangement are shown in Fig. 4. Non-identical complexes are stacked on top of each other along the *b* axis. Perchlorate *C* sits between the *A* and *B* complexes and is involved in six C—H···O interactions with H···O distance < 2.7 Å, five out of which have >CH_2_ donors. The perchlorate ions *F* and *G* interact primarily with unidentified disordered solvent molecules inside large, but isolated pockets. Amide H-atoms on N1*A* and N1*B* are donated to perchlorate ion *D* and *E*, respectively, to give one normal hydrogen bond and one three-centre hydrogen bond as listed in Table 1.

There are five other structures with a similar hydrocarbon skeleton in the Cambridge Structural Database (version 5.33 of November 2011; Allen 2002) with refcodes CECDOE (Marlin *et al.*, 2006), CECDUK (Marlin *et al.*, 2006), GUQRUG (Xu *et al.*, 2010*b*) XIXWAD (Patten *et al.*, 2008) and YUTTEN (Xu *et al.*, 2010*a*). In four complexes the substituted *N*-phenylacetamide acts as a tetradentate ligand for a metal ion (the fifth is CECDOE where the amid carbonyl group is not coordinated), but the total coordination number is always five. YUTTEN is chemically very similar to I; the metal ion is Zn^2+^ with acetonitrile as an additional ligand, only the two methyl groups on the *N*-phenyl ring are missing. The molecular conformation is almost identical to molecule I *B* except for a slightly different orientation for the *N*-phenyl group.

### Experimental

Colourless needles of (I) were obtained by dissolving 50 mg of a 1:1 mixture of the ligand and zinc perchlorate in 1 ml dry acetonitrile and transferring 0.5 ml into a 1.5 ml vial which was capped and a pinhole (0.5 mm) made in the cap. This was placed in a 5 ml vial with diethyl ether to allow slow evaporation of ether into the acetonitrile solution. After approximately 48 h at 4 °C, clear crystals appeared.

### Refinement

H atoms were positioned with idealized geometry and fixed C/N—H distances for NH, CH_3_, CH_2_ and CH (*sp*^2^) at 0.88, 0.98, 0.99 and 0.95 Å, respectively. Two perchlorate anions are located on (F) or close to (G) a twofold rotation axis, and associated atoms have an occupancy of 0.5. Furthermore, displacement ellipsoids for O atoms are large, making an unrestrained refinement difficult. Cl—O distances were thus restrained to be close to 1.43 Å through *SHELX DFIX* 1.430 0.005 commands. Electron density in the solvent regions of the crystal were initially modelled by 8 - 10 partially occupied oxygen atoms, but this proved not to be satisfactory. The electron density was consequently synthetically removed by the SQEEZE routine of the *PLATON* program (Spek, 2009), yielding a significant improvement for *R*(*F*) as well as *wR*(*F*^2^).

### Crystal data

**Table:**

[Zn(C_22_H_24_N_4_O)(C_2_H_3_N)][Zn(ClO_4_)(C_22_H_24_N_4_O)(C_2_H_3_N)](ClO_4_)_3_	*F*(000) = 5716
*M**_r_* = 1331.59	*D*_x_ = 1.490 Mg m^−^^3^
Monoclinic, *C*2/*c*	Mo *K*α radiation, λ = 0.71073 Å
Hall symbol: -C 2yc	Cell parameters from 8118 reflections
*a* = 41.253 (8) Å	θ = 2.4–25.0°
*b* = 15.057 (3) Å	µ = 1.03 mm^−^^1^
*c* = 20.809 (4) Å	*T* = 105 K
β = 106.106 (2)°	Needle, colourless
*V* = 12418 (4) Å^3^	0.91 × 0.29 × 0.22 mm
*Z* = 8	

### Data collection

**Table:**

Bruker APEXII CCD diffractometer	10987 independent reflections
Radiation source: fine-focus sealed tube	8503 reflections with *I* > 2σ(*I*)
Graphite monochromator	*R*_int_ = 0.051
Detector resolution: 8.3 pixels mm^-1^	θ_max_ = 25.1°, θ_min_ = 1.7°
Sets of exposures each taken over 0.5° ω rotation scans	*h* = −44→49
Absorption correction: multi-scan (*SADABS*; Bruker, 2007)	*k* = −17→17
*T*_min_ = 0.531, *T*_max_ = 0.798	*l* = −24→24
43906 measured reflections	

### Refinement

**Table:**

Refinement on *F*^2^	Primary atom site location: structure-invariant direct methods
Least-squares matrix: full	Secondary atom site location: difference Fourier map
*R*[*F*^2^ > 2σ(*F*^2^)] = 0.045	Hydrogen site location: inferred from neighbouring sites
*wR*(*F*^2^) = 0.124	H-atom parameters constrained
*S* = 1.07	*w* = 1/[σ^2^(*F*_o_^2^) + (0.0725*P*)^2^ + 5.6036*P*] where *P* = (*F*_o_^2^ + 2*F*_c_^2^)/3
10987 reflections	(Δ/σ)_max_ = 0.002
782 parameters	Δρ_max_ = 0.89 e Å^−^^3^
8 restraints	Δρ_min_ = −0.66 e Å^−^^3^

### Special details

**Table:**

Geometry. All e.s.d.'s (except the e.s.d. in the dihedral angle between two l.s. planes) are estimated using the full covariance matrix. The cell e.s.d.'s are taken into account individually in the estimation of e.s.d.'s in distances, angles and torsion angles; correlations between e.s.d.'s in cell parameters are only used when they are defined by crystal symmetry. An approximate (isotropic) treatment of cell e.s.d.'s is used for estimating e.s.d.'s involving l.s. planes.
Refinement. Refinement of *F*^2^ against ALL reflections. Electron density from disordered solvent removed by the *PLATON* SQUEEZE routine. Total void volume is approximately 11% of the unit cell volume.

### Fractional atomic coordinates and isotropic or equivalent isotropic displacement parameters (Å^2^)

**Table:**

	*x*	*y*	*z*	*U*_iso_*/*U*_eq_	Occ. (<1)
Zn1A	0.853323 (9)	0.59127 (2)	0.162884 (16)	0.02556 (11)	
O1A	0.86383 (6)	0.64960 (14)	0.25739 (10)	0.0357 (6)	
N1A	0.85940 (7)	0.63530 (18)	0.36241 (13)	0.0351 (7)	
H1A	0.8503	0.6055	0.3894	0.042*	
N2A	0.82241 (7)	0.50432 (18)	0.21004 (12)	0.0319 (6)	
N3A	0.80643 (7)	0.64892 (18)	0.12842 (12)	0.0301 (6)	
N4A	0.88783 (7)	0.49269 (16)	0.20125 (11)	0.0277 (6)	
N5A	0.88304 (7)	0.67580 (17)	0.12576 (12)	0.0279 (6)	
C1A	0.88254 (10)	0.7057 (2)	0.39009 (15)	0.0391 (9)	
C2A	0.86923 (10)	0.7820 (2)	0.41226 (15)	0.0411 (9)	
C3A	0.89170 (12)	0.8510 (3)	0.43869 (17)	0.0525 (11)	
H3A	0.8836	0.9036	0.4542	0.063*	
C4A	0.92540 (13)	0.8436 (3)	0.44246 (19)	0.0620 (13)	
H4A	0.9402	0.8913	0.4602	0.074*	
C5A	0.93797 (12)	0.7677 (3)	0.42077 (19)	0.0594 (12)	
H5A	0.9614	0.7637	0.4241	0.071*	
C6A	0.91669 (11)	0.6969 (3)	0.39398 (18)	0.0484 (10)	
C7A	0.83236 (10)	0.7897 (2)	0.40674 (17)	0.0465 (10)	
H71A	0.8279	0.8470	0.4251	0.070*	
H72A	0.8256	0.7414	0.4319	0.070*	
H73A	0.8194	0.7858	0.3596	0.070*	
C8A	0.93093 (11)	0.6146 (3)	0.3702 (2)	0.0627 (13)	
H81A	0.9127	0.5722	0.3521	0.094*	
H82A	0.9477	0.5874	0.4079	0.094*	
H83A	0.9416	0.6309	0.3353	0.094*	
C9A	0.85096 (9)	0.6129 (2)	0.29855 (15)	0.0322 (8)	
C10A	0.82307 (9)	0.5447 (2)	0.27544 (16)	0.0363 (8)	
H10A	0.8262	0.4972	0.3095	0.044*	
H9A	0.8011	0.5736	0.2718	0.044*	
C11A	0.78842 (9)	0.5068 (2)	0.16266 (16)	0.0394 (9)	
H11A	0.7715	0.4891	0.1859	0.047*	
H12A	0.7872	0.4640	0.1260	0.047*	
C12A	0.78053 (9)	0.5981 (2)	0.13426 (16)	0.0360 (8)	
C13A	0.80065 (9)	0.7317 (2)	0.10342 (15)	0.0358 (8)	
H13A	0.8192	0.7668	0.0997	0.043*	
C14A	0.76876 (10)	0.7668 (3)	0.08320 (17)	0.0482 (10)	
H14A	0.7652	0.8259	0.0669	0.058*	
C15A	0.74182 (11)	0.7138 (3)	0.08713 (19)	0.0599 (12)	
H15A	0.7194	0.7356	0.0720	0.072*	
C16A	0.74785 (10)	0.6298 (3)	0.11291 (18)	0.0512 (10)	
H16A	0.7296	0.5933	0.1161	0.061*	
C17A	0.83855 (9)	0.4168 (2)	0.21700 (16)	0.0343 (8)	
H17A	0.8288	0.3819	0.1758	0.041*	
H18A	0.8336	0.3848	0.2548	0.041*	
C18A	0.87640 (9)	0.4226 (2)	0.22913 (15)	0.0314 (8)	
C19A	0.92118 (8)	0.4992 (2)	0.20791 (15)	0.0300 (7)	
H19A	0.9293	0.5486	0.1886	0.036*	
C20A	0.94391 (10)	0.4368 (2)	0.24175 (17)	0.0407 (9)	
H20A	0.9673	0.4430	0.2454	0.049*	
C21A	0.93244 (11)	0.3651 (2)	0.27029 (18)	0.0458 (10)	
H21A	0.9477	0.3210	0.2936	0.055*	
C22A	0.89839 (11)	0.3586 (2)	0.26436 (16)	0.0410 (9)	
H22A	0.8900	0.3103	0.2844	0.049*	
C23A	0.90154 (8)	0.7206 (2)	0.11036 (14)	0.0298 (7)	
C24A	0.92560 (10)	0.7789 (3)	0.09111 (17)	0.0472 (10)	
H24A	0.9193	0.8410	0.0951	0.071*	
H25A	0.9253	0.7667	0.0447	0.071*	
H26A	0.9483	0.7682	0.1206	0.071*	
Zn1B	0.850909 (9)	0.10724 (2)	0.182215 (16)	0.02359 (11)	
O1B	0.86112 (5)	0.16804 (13)	0.27244 (9)	0.0280 (5)	
N1B	0.86223 (7)	0.15562 (16)	0.38094 (12)	0.0285 (6)	
H1B	0.8530	0.1312	0.4101	0.034*	
N2B	0.81877 (7)	0.02712 (17)	0.23148 (12)	0.0283 (6)	
N3B	0.80573 (7)	0.12094 (17)	0.11358 (12)	0.0266 (6)	
N4B	0.88036 (7)	−0.00293 (17)	0.20878 (11)	0.0295 (6)	
N5B	0.88183 (7)	0.18507 (17)	0.14441 (12)	0.0298 (6)	
C1B	0.88784 (9)	0.2221 (2)	0.40386 (14)	0.0303 (7)	
C2B	0.87858 (9)	0.3043 (2)	0.42471 (15)	0.0331 (8)	
C3B	0.90378 (11)	0.3676 (3)	0.44669 (19)	0.0480 (10)	
H3B	0.8983	0.4240	0.4613	0.058*	
C4B	0.93672 (11)	0.3495 (3)	0.4476 (2)	0.0628 (14)	
H4B	0.9536	0.3938	0.4621	0.075*	
C5B	0.94530 (10)	0.2678 (3)	0.42749 (18)	0.0554 (12)	
H5B	0.9681	0.2560	0.4291	0.066*	
C6B	0.92113 (9)	0.2023 (2)	0.40488 (16)	0.0389 (8)	
C7B	0.84309 (9)	0.3234 (2)	0.42462 (16)	0.0396 (8)	
H71B	0.8416	0.3839	0.4409	0.059*	
H72B	0.8361	0.2810	0.4539	0.059*	
H73B	0.8283	0.3177	0.3790	0.059*	
C8B	0.93100 (11)	0.1128 (3)	0.3822 (2)	0.0536 (11)	
H81B	0.9109	0.0750	0.3677	0.080*	
H82B	0.9476	0.0842	0.4193	0.080*	
H83B	0.9408	0.1215	0.3447	0.080*	
C9B	0.85185 (8)	0.12953 (19)	0.31777 (14)	0.0264 (7)	
C10B	0.82974 (9)	0.0476 (2)	0.30370 (14)	0.0336 (8)	
H10B	0.8424	−0.0036	0.3283	0.040*	
H9B	0.8096	0.0573	0.3200	0.040*	
C11B	0.78361 (9)	0.0504 (2)	0.19832 (16)	0.0355 (8)	
H11B	0.7762	0.0973	0.2245	0.043*	
H12B	0.7692	−0.0023	0.1974	0.043*	
C12B	0.77897 (8)	0.0831 (2)	0.12761 (15)	0.0304 (7)	
C13B	0.80221 (9)	0.1555 (2)	0.05235 (14)	0.0305 (7)	
H13B	0.8212	0.1819	0.0427	0.037*	
C14B	0.77192 (9)	0.1537 (2)	0.00329 (16)	0.0392 (8)	
H14B	0.7699	0.1788	−0.0395	0.047*	
C15B	0.74445 (9)	0.1144 (3)	0.01802 (18)	0.0443 (9)	
H15B	0.7232	0.1120	−0.0148	0.053*	
C16B	0.74821 (9)	0.0789 (2)	0.08056 (17)	0.0401 (9)	
H16B	0.7296	0.0515	0.0911	0.048*	
C17B	0.82742 (9)	−0.0648 (2)	0.21670 (15)	0.0341 (8)	
H17B	0.8152	−0.0801	0.1700	0.041*	
H18B	0.8203	−0.1068	0.2467	0.041*	
C18B	0.86462 (10)	−0.0734 (2)	0.22624 (14)	0.0347 (8)	
C19B	0.91337 (9)	−0.0069 (2)	0.21427 (15)	0.0383 (8)	
H19B	0.9241	0.0431	0.2012	0.046*	
C20B	0.93232 (12)	−0.0811 (3)	0.23819 (19)	0.0541 (11)	
H20B	0.9558	−0.0819	0.2424	0.065*	
C21B	0.91691 (13)	−0.1536 (3)	0.25579 (19)	0.0604 (13)	
H21B	0.9295	−0.2057	0.2717	0.072*	
C22B	0.88279 (13)	−0.1505 (2)	0.25024 (17)	0.0515 (11)	
H22B	0.8718	−0.2004	0.2626	0.062*	
C23B	0.90020 (8)	0.2236 (2)	0.12319 (15)	0.0281 (7)	
C24B	0.92369 (9)	0.2734 (2)	0.09660 (17)	0.0398 (8)	
H24B	0.9197	0.3372	0.1000	0.060*	
H25B	0.9204	0.2575	0.0496	0.060*	
H26B	0.9469	0.2593	0.1222	0.060*	
Cl1C	0.75770 (2)	0.28630 (6)	0.23363 (4)	0.0396 (2)	
O1C	0.75333 (10)	0.3701 (2)	0.25924 (17)	0.0862 (12)	
O2C	0.78840 (7)	0.24711 (19)	0.27441 (14)	0.0571 (7)	
O3C	0.73026 (8)	0.2301 (2)	0.23552 (16)	0.0809 (11)	
O4C	0.76038 (7)	0.29391 (18)	0.16670 (12)	0.0530 (7)	
Cl1D	0.850080 (19)	0.44683 (5)	0.03225 (3)	0.02583 (17)	
O1D	0.83426 (6)	0.51464 (14)	0.06334 (10)	0.0336 (5)	
O2D	0.88496 (6)	0.46675 (16)	0.04288 (11)	0.0383 (6)	
O3D	0.83300 (6)	0.44678 (15)	−0.03836 (10)	0.0338 (5)	
O4D	0.84614 (6)	0.36250 (15)	0.06091 (11)	0.0381 (6)	
Cl1E	0.83903 (2)	−0.05427 (5)	0.02203 (4)	0.0377 (2)	
O1E	0.86080 (8)	0.0128 (2)	0.05791 (13)	0.0635 (8)	
O2E	0.80982 (8)	−0.06216 (17)	0.04704 (13)	0.0554 (8)	
O3E	0.85568 (9)	−0.13788 (19)	0.02709 (15)	0.0730 (10)	
O4E	0.82850 (6)	−0.02999 (15)	−0.04755 (10)	0.0376 (6)	
Cl1F	1.0000	0.70818 (12)	0.2500	0.0630 (4)	
O1F	0.99954 (17)	0.7857 (4)	0.2109 (4)	0.091 (3)	0.50
O2F	0.9785 (3)	0.6454 (9)	0.2089 (7)	0.201 (9)	0.50
O3F	0.9906 (3)	0.7262 (9)	0.3106 (4)	0.169 (6)	0.50
O4F	1.03261 (14)	0.6715 (6)	0.2706 (4)	0.0487 (17)	0.50
Cl1G	0.9993 (2)	0.18690 (15)	0.2590 (3)	0.0619 (13)	0.50
O1G	0.9975 (2)	0.2081 (7)	0.3251 (3)	0.099 (3)	0.50
O2G	0.9977 (2)	0.2677 (5)	0.2198 (4)	0.102 (3)	0.50
O3G	1.0325 (2)	0.1570 (7)	0.2653 (4)	0.058 (2)	0.50
O4G	0.9744 (4)	0.1271 (10)	0.2236 (9)	0.177 (9)	0.50

### Atomic displacement parameters (Å^2^)

**Table:**

	*U*^11^	*U*^22^	*U*^33^	*U*^12^	*U*^13^	*U*^23^
Zn1A	0.0336 (2)	0.0267 (2)	0.02145 (19)	−0.00585 (15)	0.01601 (15)	−0.00028 (13)
O1A	0.0585 (16)	0.0321 (12)	0.0247 (11)	−0.0157 (11)	0.0252 (11)	−0.0058 (9)
N1A	0.0521 (19)	0.0370 (16)	0.0237 (14)	−0.0075 (13)	0.0230 (13)	−0.0007 (11)
N2A	0.0405 (17)	0.0357 (15)	0.0262 (13)	−0.0123 (13)	0.0205 (12)	−0.0069 (11)
N3A	0.0319 (16)	0.0360 (16)	0.0268 (14)	−0.0038 (12)	0.0155 (12)	−0.0065 (11)
N4A	0.0405 (17)	0.0247 (13)	0.0204 (12)	−0.0064 (12)	0.0124 (11)	0.0003 (10)
N5A	0.0360 (16)	0.0282 (14)	0.0220 (13)	−0.0023 (12)	0.0123 (12)	0.0038 (10)
C1A	0.059 (3)	0.045 (2)	0.0185 (15)	−0.0104 (18)	0.0196 (16)	−0.0038 (14)
C2A	0.064 (3)	0.043 (2)	0.0184 (15)	−0.0044 (18)	0.0158 (16)	−0.0033 (14)
C3A	0.080 (3)	0.050 (2)	0.0283 (19)	−0.011 (2)	0.017 (2)	−0.0108 (16)
C4A	0.084 (4)	0.066 (3)	0.037 (2)	−0.032 (3)	0.018 (2)	−0.021 (2)
C5A	0.068 (3)	0.077 (3)	0.039 (2)	−0.024 (2)	0.024 (2)	−0.022 (2)
C6A	0.066 (3)	0.053 (2)	0.0343 (19)	−0.014 (2)	0.0269 (19)	−0.0153 (17)
C7A	0.069 (3)	0.043 (2)	0.0305 (18)	0.0051 (19)	0.0178 (18)	−0.0040 (15)
C8A	0.055 (3)	0.083 (3)	0.060 (3)	−0.012 (2)	0.033 (2)	−0.032 (2)
C9A	0.047 (2)	0.0301 (17)	0.0269 (17)	−0.0046 (15)	0.0227 (15)	−0.0010 (13)
C10A	0.056 (2)	0.0345 (18)	0.0282 (17)	−0.0119 (17)	0.0275 (16)	−0.0047 (14)
C11A	0.042 (2)	0.049 (2)	0.0333 (18)	−0.0195 (17)	0.0201 (16)	−0.0115 (15)
C12A	0.033 (2)	0.056 (2)	0.0238 (16)	−0.0071 (17)	0.0162 (14)	−0.0118 (15)
C13A	0.039 (2)	0.045 (2)	0.0258 (16)	0.0003 (16)	0.0148 (15)	−0.0082 (14)
C14A	0.051 (3)	0.070 (3)	0.0244 (18)	0.015 (2)	0.0121 (17)	−0.0015 (17)
C15A	0.036 (2)	0.108 (4)	0.038 (2)	0.015 (2)	0.0150 (18)	0.006 (2)
C16A	0.042 (2)	0.085 (3)	0.0302 (19)	−0.007 (2)	0.0166 (17)	0.0027 (19)
C17A	0.053 (2)	0.0292 (17)	0.0286 (17)	−0.0150 (16)	0.0246 (16)	−0.0050 (13)
C18A	0.055 (2)	0.0224 (16)	0.0210 (15)	−0.0104 (15)	0.0178 (15)	−0.0039 (12)
C19A	0.036 (2)	0.0290 (17)	0.0273 (16)	−0.0044 (14)	0.0123 (14)	0.0012 (13)
C20A	0.044 (2)	0.043 (2)	0.0370 (19)	0.0028 (17)	0.0141 (17)	0.0000 (15)
C21A	0.063 (3)	0.035 (2)	0.039 (2)	0.0108 (18)	0.0149 (19)	0.0054 (16)
C22A	0.077 (3)	0.0217 (17)	0.0294 (18)	−0.0029 (17)	0.0233 (18)	0.0018 (13)
C23A	0.035 (2)	0.0377 (18)	0.0165 (14)	−0.0063 (15)	0.0070 (13)	0.0032 (12)
C24A	0.050 (2)	0.063 (3)	0.0301 (18)	−0.027 (2)	0.0136 (17)	0.0120 (17)
Zn1B	0.0323 (2)	0.02355 (19)	0.01785 (18)	−0.00281 (15)	0.01191 (15)	0.00170 (13)
O1B	0.0390 (13)	0.0292 (12)	0.0199 (10)	−0.0083 (10)	0.0148 (9)	−0.0004 (8)
N1B	0.0420 (17)	0.0286 (14)	0.0200 (12)	−0.0082 (12)	0.0171 (12)	−0.0026 (10)
N2B	0.0417 (17)	0.0279 (14)	0.0186 (12)	−0.0111 (12)	0.0140 (11)	−0.0053 (10)
N3B	0.0317 (16)	0.0276 (13)	0.0244 (13)	−0.0009 (11)	0.0140 (11)	−0.0030 (10)
N4B	0.0459 (18)	0.0264 (14)	0.0195 (12)	0.0023 (12)	0.0143 (12)	0.0006 (10)
N5B	0.0361 (17)	0.0309 (14)	0.0248 (13)	−0.0018 (13)	0.0126 (12)	0.0026 (11)
C1B	0.041 (2)	0.0364 (18)	0.0170 (14)	−0.0105 (15)	0.0134 (13)	−0.0042 (12)
C2B	0.048 (2)	0.0337 (18)	0.0227 (15)	−0.0107 (16)	0.0191 (15)	−0.0046 (13)
C3B	0.067 (3)	0.043 (2)	0.045 (2)	−0.023 (2)	0.033 (2)	−0.0189 (17)
C4B	0.059 (3)	0.086 (3)	0.056 (3)	−0.043 (2)	0.037 (2)	−0.039 (2)
C5B	0.043 (2)	0.092 (3)	0.038 (2)	−0.022 (2)	0.0228 (18)	−0.029 (2)
C6B	0.042 (2)	0.053 (2)	0.0247 (16)	−0.0063 (18)	0.0153 (15)	−0.0099 (15)
C7B	0.054 (2)	0.0364 (19)	0.0300 (18)	−0.0040 (17)	0.0149 (16)	−0.0051 (14)
C8B	0.050 (3)	0.068 (3)	0.043 (2)	0.008 (2)	0.0126 (19)	−0.0159 (19)
C9B	0.0375 (19)	0.0229 (15)	0.0231 (15)	−0.0012 (13)	0.0157 (14)	−0.0019 (12)
C10B	0.055 (2)	0.0329 (18)	0.0181 (15)	−0.0144 (16)	0.0191 (15)	−0.0053 (12)
C11B	0.041 (2)	0.040 (2)	0.0337 (18)	−0.0138 (16)	0.0234 (16)	−0.0060 (14)
C12B	0.0316 (19)	0.0346 (18)	0.0289 (17)	−0.0041 (14)	0.0148 (14)	−0.0095 (13)
C13B	0.038 (2)	0.0323 (18)	0.0237 (16)	0.0019 (14)	0.0118 (14)	0.0011 (13)
C14B	0.042 (2)	0.049 (2)	0.0258 (17)	0.0093 (17)	0.0066 (15)	0.0006 (15)
C15B	0.030 (2)	0.062 (3)	0.037 (2)	0.0012 (18)	0.0034 (16)	−0.0092 (17)
C16B	0.034 (2)	0.052 (2)	0.038 (2)	−0.0043 (17)	0.0157 (16)	−0.0107 (16)
C17B	0.062 (3)	0.0224 (16)	0.0216 (16)	−0.0105 (15)	0.0183 (16)	−0.0029 (12)
C18B	0.068 (3)	0.0233 (16)	0.0142 (14)	−0.0010 (16)	0.0135 (15)	−0.0031 (12)
C19B	0.050 (2)	0.040 (2)	0.0276 (17)	0.0092 (17)	0.0155 (16)	−0.0012 (14)
C20B	0.072 (3)	0.054 (3)	0.041 (2)	0.031 (2)	0.023 (2)	0.0041 (18)
C21B	0.100 (4)	0.049 (3)	0.036 (2)	0.037 (3)	0.025 (2)	0.0056 (18)
C22B	0.105 (4)	0.0265 (19)	0.0273 (18)	0.008 (2)	0.025 (2)	0.0015 (14)
C23B	0.0345 (19)	0.0276 (17)	0.0240 (15)	0.0004 (14)	0.0112 (14)	0.0011 (12)
C24B	0.040 (2)	0.048 (2)	0.0365 (19)	−0.0099 (17)	0.0182 (16)	0.0063 (15)
Cl1C	0.0416 (5)	0.0407 (5)	0.0386 (5)	−0.0035 (4)	0.0145 (4)	0.0062 (4)
O1C	0.147 (4)	0.0483 (19)	0.095 (3)	0.0210 (19)	0.086 (3)	0.0033 (16)
O2C	0.0470 (17)	0.0647 (19)	0.0505 (16)	0.0011 (14)	−0.0019 (13)	0.0012 (14)
O3C	0.0491 (19)	0.109 (3)	0.074 (2)	−0.0276 (18)	−0.0015 (16)	0.0512 (19)
O4C	0.0686 (19)	0.0579 (17)	0.0348 (14)	−0.0013 (14)	0.0182 (13)	0.0032 (12)
Cl1D	0.0305 (4)	0.0283 (4)	0.0233 (4)	−0.0013 (3)	0.0151 (3)	−0.0022 (3)
O1D	0.0405 (14)	0.0354 (13)	0.0281 (11)	0.0048 (10)	0.0151 (10)	−0.0096 (9)
O2D	0.0308 (14)	0.0494 (15)	0.0392 (13)	−0.0029 (11)	0.0174 (11)	0.0063 (11)
O3D	0.0397 (14)	0.0441 (14)	0.0208 (11)	0.0025 (11)	0.0138 (10)	−0.0056 (9)
O4D	0.0473 (16)	0.0306 (12)	0.0406 (13)	−0.0016 (11)	0.0191 (11)	0.0043 (10)
Cl1E	0.0660 (6)	0.0328 (4)	0.0176 (4)	0.0110 (4)	0.0173 (4)	0.0008 (3)
O1E	0.075 (2)	0.076 (2)	0.0349 (15)	−0.0066 (17)	0.0065 (14)	−0.0191 (14)
O2E	0.097 (2)	0.0415 (15)	0.0466 (15)	0.0042 (15)	0.0510 (16)	−0.0005 (12)
O3E	0.116 (3)	0.0520 (18)	0.0602 (19)	0.0424 (18)	0.0406 (19)	0.0181 (14)
O4E	0.0511 (16)	0.0474 (14)	0.0159 (10)	0.0017 (12)	0.0122 (10)	0.0028 (9)
Cl1F	0.0300 (8)	0.0834 (12)	0.0714 (10)	0.000	0.0072 (7)	0.000
O1F	0.048 (4)	0.065 (4)	0.136 (7)	−0.019 (4)	−0.012 (5)	0.066 (4)
O2F	0.128 (11)	0.200 (17)	0.201 (17)	−0.126 (12)	−0.077 (10)	0.096 (12)
O3F	0.164 (11)	0.202 (13)	0.201 (12)	0.081 (10)	0.150 (10)	0.073 (10)
O4F	0.034 (4)	0.062 (4)	0.049 (3)	0.014 (3)	0.011 (3)	−0.003 (3)
Cl1G	0.0392 (11)	0.0748 (12)	0.076 (4)	−0.012 (2)	0.024 (2)	−0.030 (2)
O1G	0.088 (6)	0.144 (8)	0.080 (5)	0.025 (5)	0.047 (4)	−0.008 (5)
O2G	0.077 (5)	0.081 (5)	0.155 (10)	−0.002 (5)	0.043 (8)	0.048 (5)
O3G	0.046 (5)	0.078 (6)	0.051 (4)	0.018 (4)	0.016 (3)	−0.002 (3)
O4G	0.103 (11)	0.122 (12)	0.262 (19)	−0.064 (10)	−0.020 (10)	−0.007 (10)

### Geometric parameters (Å, º)

**Table:**

Zn1A—N4A	2.058 (3)	N2B—C10B	1.477 (4)
Zn1A—N3A	2.059 (3)	N2B—C17B	1.483 (4)
Zn1A—N5A	2.060 (3)	N3B—C12B	1.344 (4)
Zn1A—O1A	2.087 (2)	N3B—C13B	1.346 (4)
Zn1A—N2A	2.236 (2)	N4B—C19B	1.336 (4)
Zn1A—O1D	2.310 (2)	N4B—C18B	1.346 (4)
O1A—C9A	1.254 (4)	N5B—C23B	1.137 (4)
N1A—C9A	1.321 (4)	C1B—C6B	1.400 (5)
N1A—C1A	1.435 (4)	C1B—C2B	1.400 (4)
N1A—H1A	0.8800	C2B—C3B	1.391 (5)
N2A—C17A	1.466 (4)	C2B—C7B	1.492 (5)
N2A—C11A	1.474 (4)	C3B—C4B	1.381 (6)
N2A—C10A	1.484 (4)	C3B—H3B	0.9500
N3A—C13A	1.346 (4)	C4B—C5B	1.377 (6)
N3A—C12A	1.347 (4)	C4B—H4B	0.9500
N4A—C19A	1.347 (4)	C5B—C6B	1.388 (5)
N4A—C18A	1.351 (4)	C5B—H5B	0.9500
N5A—C23A	1.129 (4)	C6B—C8B	1.521 (5)
C1A—C6A	1.395 (5)	C7B—H71B	0.9800
C1A—C2A	1.406 (5)	C7B—H72B	0.9800
C2A—C3A	1.399 (5)	C7B—H73B	0.9800
C2A—C7A	1.498 (5)	C8B—H81B	0.9800
C3A—C4A	1.375 (6)	C8B—H82B	0.9800
C3A—H3A	0.9500	C8B—H83B	0.9800
C4A—C5A	1.381 (6)	C9B—C10B	1.513 (4)
C4A—H4A	0.9500	C10B—H10B	0.9900
C5A—C6A	1.396 (5)	C10B—H9B	0.9900
C5A—H5A	0.9500	C11B—C12B	1.513 (4)
C6A—C8A	1.512 (6)	C11B—H11B	0.9900
C7A—H71A	0.9800	C11B—H12B	0.9900
C7A—H72A	0.9800	C12B—C16B	1.372 (5)
C7A—H73A	0.9800	C13B—C14B	1.377 (5)
C8A—H81A	0.9800	C13B—H13B	0.9500
C8A—H82A	0.9800	C14B—C15B	1.386 (5)
C8A—H83A	0.9800	C14B—H14B	0.9500
C9A—C10A	1.517 (5)	C15B—C16B	1.376 (5)
C10A—H10A	0.9900	C15B—H15B	0.9500
C10A—H9A	0.9900	C16B—H16B	0.9500
C11A—C12A	1.496 (5)	C17B—C18B	1.497 (5)
C11A—H11A	0.9900	C17B—H17B	0.9900
C11A—H12A	0.9900	C17B—H18B	0.9900
C12A—C16A	1.382 (5)	C18B—C22B	1.396 (5)
C13A—C14A	1.371 (5)	C19B—C20B	1.375 (5)
C13A—H13A	0.9500	C19B—H19B	0.9500
C14A—C15A	1.389 (6)	C20B—C21B	1.363 (6)
C14A—H14A	0.9500	C20B—H20B	0.9500
C15A—C16A	1.369 (6)	C21B—C22B	1.381 (6)
C15A—H15A	0.9500	C21B—H21B	0.9500
C16A—H16A	0.9500	C22B—H22B	0.9500
C17A—C18A	1.514 (5)	C23B—C24B	1.451 (4)
C17A—H17A	0.9900	C24B—H24B	0.9800
C17A—H18A	0.9900	C24B—H25B	0.9800
C18A—C22A	1.386 (5)	C24B—H26B	0.9800
C19A—C20A	1.376 (5)	Cl1C—O1C	1.401 (3)
C19A—H19A	0.9500	Cl1C—O3C	1.422 (3)
C20A—C21A	1.378 (5)	Cl1C—O4C	1.432 (3)
C20A—H20A	0.9500	Cl1C—O2C	1.441 (3)
C21A—C22A	1.379 (5)	Cl1D—O2D	1.426 (2)
C21A—H21A	0.9500	Cl1D—O4D	1.431 (2)
C22A—H22A	0.9500	Cl1D—O3D	1.443 (2)
C23A—C24A	1.462 (4)	Cl1D—O1D	1.456 (2)
C24A—H24A	0.9800	Cl1E—O1E	1.418 (3)
C24A—H25A	0.9800	Cl1E—O3E	1.424 (3)
C24A—H26A	0.9800	Cl1E—O4E	1.439 (2)
Zn1B—N3B	2.020 (3)	Cl1E—O2E	1.444 (3)
Zn1B—O1B	2.025 (2)	Cl1F—O4F	1.406 (4)
Zn1B—N4B	2.040 (3)	Cl1F—O2F	1.411 (5)
Zn1B—N5B	2.043 (3)	Cl1F—O1F	1.420 (4)
Zn1B—N2B	2.240 (2)	Cl1F—O3F	1.443 (5)
O1B—C9B	1.254 (3)	Cl1G—O3G	1.410 (5)
N1B—C9B	1.324 (4)	Cl1G—O4G	1.412 (5)
N1B—C1B	1.437 (4)	Cl1G—O1G	1.436 (5)
N1B—H1B	0.8800	Cl1G—O2G	1.456 (4)
N2B—C11B	1.466 (4)		
			
N4A—Zn1A—N3A	157.06 (10)	C1B—N1B—H1B	118.6
N4A—Zn1A—N5A	99.87 (10)	C11B—N2B—C10B	114.4 (3)
N3A—Zn1A—N5A	102.02 (10)	C11B—N2B—C17B	112.9 (3)
N4A—Zn1A—O1A	89.86 (9)	C10B—N2B—C17B	112.5 (2)
N3A—Zn1A—O1A	94.60 (10)	C11B—N2B—Zn1B	106.84 (18)
N5A—Zn1A—O1A	95.95 (9)	C10B—N2B—Zn1B	107.45 (18)
N4A—Zn1A—N2A	79.92 (10)	C17B—N2B—Zn1B	101.59 (18)
N3A—Zn1A—N2A	78.72 (10)	C12B—N3B—C13B	119.3 (3)
N5A—Zn1A—N2A	176.00 (9)	C12B—N3B—Zn1B	117.1 (2)
O1A—Zn1A—N2A	80.06 (9)	C13B—N3B—Zn1B	123.0 (2)
N4A—Zn1A—O1D	90.82 (9)	C19B—N4B—C18B	119.9 (3)
N3A—Zn1A—O1D	81.08 (9)	C19B—N4B—Zn1B	125.2 (2)
N5A—Zn1A—O1D	93.57 (9)	C18B—N4B—Zn1B	114.8 (2)
O1A—Zn1A—O1D	170.19 (8)	C23B—N5B—Zn1B	175.6 (3)
N2A—Zn1A—O1D	90.42 (9)	C6B—C1B—C2B	122.3 (3)
C9A—O1A—Zn1A	116.2 (2)	C6B—C1B—N1B	119.0 (3)
C9A—N1A—C1A	123.5 (3)	C2B—C1B—N1B	118.7 (3)
C9A—N1A—H1A	118.2	C3B—C2B—C1B	117.7 (3)
C1A—N1A—H1A	118.2	C3B—C2B—C7B	120.8 (3)
C17A—N2A—C11A	114.3 (3)	C1B—C2B—C7B	121.5 (3)
C17A—N2A—C10A	112.7 (3)	C4B—C3B—C2B	120.8 (4)
C11A—N2A—C10A	112.0 (3)	C4B—C3B—H3B	119.6
C17A—N2A—Zn1A	105.74 (18)	C2B—C3B—H3B	119.6
C11A—N2A—Zn1A	104.13 (19)	C5B—C4B—C3B	120.5 (4)
C10A—N2A—Zn1A	107.15 (18)	C5B—C4B—H4B	119.8
C13A—N3A—C12A	120.1 (3)	C3B—C4B—H4B	119.8
C13A—N3A—Zn1A	125.0 (2)	C4B—C5B—C6B	121.1 (4)
C12A—N3A—Zn1A	114.9 (2)	C4B—C5B—H5B	119.4
C19A—N4A—C18A	118.7 (3)	C6B—C5B—H5B	119.4
C19A—N4A—Zn1A	124.3 (2)	C5B—C6B—C1B	117.6 (3)
C18A—N4A—Zn1A	116.3 (2)	C5B—C6B—C8B	120.4 (3)
C23A—N5A—Zn1A	173.9 (3)	C1B—C6B—C8B	122.0 (3)
C6A—C1A—C2A	122.6 (3)	C2B—C7B—H71B	109.5
C6A—C1A—N1A	120.1 (3)	C2B—C7B—H72B	109.5
C2A—C1A—N1A	117.3 (3)	H71B—C7B—H72B	109.5
C3A—C2A—C1A	117.4 (4)	C2B—C7B—H73B	109.5
C3A—C2A—C7A	121.4 (3)	H71B—C7B—H73B	109.5
C1A—C2A—C7A	121.2 (3)	H72B—C7B—H73B	109.5
C4A—C3A—C2A	120.7 (4)	C6B—C8B—H81B	109.5
C4A—C3A—H3A	119.6	C6B—C8B—H82B	109.5
C2A—C3A—H3A	119.6	H81B—C8B—H82B	109.5
C3A—C4A—C5A	120.9 (4)	C6B—C8B—H83B	109.5
C3A—C4A—H4A	119.5	H81B—C8B—H83B	109.5
C5A—C4A—H4A	119.5	H82B—C8B—H83B	109.5
C4A—C5A—C6A	120.7 (4)	O1B—C9B—N1B	121.8 (3)
C4A—C5A—H5A	119.6	O1B—C9B—C10B	121.6 (3)
C6A—C5A—H5A	119.6	N1B—C9B—C10B	116.4 (2)
C1A—C6A—C5A	117.6 (4)	N2B—C10B—C9B	111.4 (2)
C1A—C6A—C8A	122.4 (3)	N2B—C10B—H10B	109.3
C5A—C6A—C8A	119.9 (4)	C9B—C10B—H10B	109.3
C2A—C7A—H71A	109.5	N2B—C10B—H9B	109.3
C2A—C7A—H72A	109.5	C9B—C10B—H9B	109.3
H71A—C7A—H72A	109.5	H10B—C10B—H9B	108.0
C2A—C7A—H73A	109.5	N2B—C11B—C12B	112.2 (2)
H71A—C7A—H73A	109.5	N2B—C11B—H11B	109.2
H72A—C7A—H73A	109.5	C12B—C11B—H11B	109.2
C6A—C8A—H81A	109.5	N2B—C11B—H12B	109.2
C6A—C8A—H82A	109.5	C12B—C11B—H12B	109.2
H81A—C8A—H82A	109.5	H11B—C11B—H12B	107.9
C6A—C8A—H83A	109.5	N3B—C12B—C16B	121.2 (3)
H81A—C8A—H83A	109.5	N3B—C12B—C11B	117.3 (3)
H82A—C8A—H83A	109.5	C16B—C12B—C11B	121.4 (3)
O1A—C9A—N1A	122.1 (3)	N3B—C13B—C14B	122.1 (3)
O1A—C9A—C10A	120.6 (3)	N3B—C13B—H13B	118.9
N1A—C9A—C10A	117.2 (3)	C14B—C13B—H13B	118.9
N2A—C10A—C9A	112.8 (2)	C13B—C14B—C15B	118.3 (3)
N2A—C10A—H10A	109.0	C13B—C14B—H14B	120.9
C9A—C10A—H10A	109.0	C15B—C14B—H14B	120.9
N2A—C10A—H9A	109.0	C16B—C15B—C14B	119.4 (3)
C9A—C10A—H9A	109.0	C16B—C15B—H15B	120.3
H10A—C10A—H9A	107.8	C14B—C15B—H15B	120.3
N2A—C11A—C12A	110.5 (3)	C12B—C16B—C15B	119.7 (3)
N2A—C11A—H11A	109.6	C12B—C16B—H16B	120.1
C12A—C11A—H11A	109.6	C15B—C16B—H16B	120.1
N2A—C11A—H12A	109.6	N2B—C17B—C18B	110.5 (3)
C12A—C11A—H12A	109.6	N2B—C17B—H17B	109.5
H11A—C11A—H12A	108.1	C18B—C17B—H17B	109.5
N3A—C12A—C16A	120.1 (4)	N2B—C17B—H18B	109.5
N3A—C12A—C11A	117.8 (3)	C18B—C17B—H18B	109.5
C16A—C12A—C11A	122.0 (3)	H17B—C17B—H18B	108.1
N3A—C13A—C14A	121.8 (4)	N4B—C18B—C22B	120.0 (4)
N3A—C13A—H13A	119.1	N4B—C18B—C17B	116.7 (3)
C14A—C13A—H13A	119.1	C22B—C18B—C17B	123.3 (3)
C13A—C14A—C15A	118.4 (4)	N4B—C19B—C20B	122.1 (4)
C13A—C14A—H14A	120.8	N4B—C19B—H19B	118.9
C15A—C14A—H14A	120.8	C20B—C19B—H19B	118.9
C16A—C15A—C14A	119.5 (4)	C21B—C20B—C19B	119.1 (4)
C16A—C15A—H15A	120.2	C21B—C20B—H20B	120.4
C14A—C15A—H15A	120.2	C19B—C20B—H20B	120.4
C15A—C16A—C12A	120.0 (4)	C20B—C21B—C22B	119.4 (4)
C15A—C16A—H16A	120.0	C20B—C21B—H21B	120.3
C12A—C16A—H16A	120.0	C22B—C21B—H21B	120.3
N2A—C17A—C18A	112.6 (3)	C21B—C22B—C18B	119.4 (4)
N2A—C17A—H17A	109.1	C21B—C22B—H22B	120.3
C18A—C17A—H17A	109.1	C18B—C22B—H22B	120.3
N2A—C17A—H18A	109.1	N5B—C23B—C24B	179.5 (4)
C18A—C17A—H18A	109.1	C23B—C24B—H24B	109.5
H17A—C17A—H18A	107.8	C23B—C24B—H25B	109.5
N4A—C18A—C22A	121.0 (3)	H24B—C24B—H25B	109.5
N4A—C18A—C17A	116.1 (3)	C23B—C24B—H26B	109.5
C22A—C18A—C17A	122.8 (3)	H24B—C24B—H26B	109.5
N4A—C19A—C20A	122.3 (3)	H25B—C24B—H26B	109.5
N4A—C19A—H19A	118.8	O1C—Cl1C—O3C	109.8 (2)
C20A—C19A—H19A	118.8	O1C—Cl1C—O4C	110.52 (18)
C19A—C20A—C21A	119.3 (4)	O3C—Cl1C—O4C	110.57 (19)
C19A—C20A—H20A	120.4	O1C—Cl1C—O2C	109.3 (2)
C21A—C20A—H20A	120.4	O3C—Cl1C—O2C	108.43 (18)
C20A—C21A—C22A	118.7 (3)	O4C—Cl1C—O2C	108.26 (17)
C20A—C21A—H21A	120.7	O2D—Cl1D—O4D	110.39 (15)
C22A—C21A—H21A	120.7	O2D—Cl1D—O3D	110.17 (13)
C21A—C22A—C18A	120.0 (3)	O4D—Cl1D—O3D	110.13 (14)
C21A—C22A—H22A	120.0	O2D—Cl1D—O1D	109.92 (14)
C18A—C22A—H22A	120.0	O4D—Cl1D—O1D	108.68 (14)
N5A—C23A—C24A	179.4 (4)	O3D—Cl1D—O1D	107.48 (13)
C23A—C24A—H24A	109.5	Cl1D—O1D—Zn1A	131.72 (14)
C23A—C24A—H25A	109.5	O1E—Cl1E—O3E	111.6 (2)
H24A—C24A—H25A	109.5	O1E—Cl1E—O4E	108.61 (16)
C23A—C24A—H26A	109.5	O3E—Cl1E—O4E	107.69 (16)
H24A—C24A—H26A	109.5	O1E—Cl1E—O2E	109.80 (17)
H25A—C24A—H26A	109.5	O3E—Cl1E—O2E	109.52 (18)
N3B—Zn1B—O1B	121.54 (9)	O4E—Cl1E—O2E	109.54 (16)
N3B—Zn1B—N4B	129.65 (10)	O4F—Cl1F—O2F	108.0 (7)
O1B—Zn1B—N4B	99.28 (9)	O4F—Cl1F—O1F	110.9 (4)
N3B—Zn1B—N5B	102.79 (10)	O2F—Cl1F—O1F	107.3 (7)
O1B—Zn1B—N5B	96.10 (9)	O4F—Cl1F—O3F	105.8 (6)
N4B—Zn1B—N5B	100.51 (11)	O2F—Cl1F—O3F	112.3 (9)
N3B—Zn1B—N2B	80.29 (10)	O1F—Cl1F—O3F	112.4 (8)
O1B—Zn1B—N2B	79.43 (8)	O3G—Cl1G—O4G	113.2 (8)
N4B—Zn1B—N2B	79.72 (10)	O3G—Cl1G—O1G	106.8 (6)
N5B—Zn1B—N2B	175.48 (9)	O4G—Cl1G—O1G	114.6 (9)
C9B—O1B—Zn1B	117.38 (19)	O3G—Cl1G—O2G	102.2 (7)
C9B—N1B—C1B	122.8 (2)	O4G—Cl1G—O2G	109.1 (10)
C9B—N1B—H1B	118.6	O1G—Cl1G—O2G	110.2 (7)
			
N4A—Zn1A—O1A—C9A	−76.9 (3)	N3B—Zn1B—O1B—C9B	84.3 (2)
N3A—Zn1A—O1A—C9A	80.6 (3)	N4B—Zn1B—O1B—C9B	−64.9 (2)
N5A—Zn1A—O1A—C9A	−176.8 (3)	N5B—Zn1B—O1B—C9B	−166.7 (2)
N2A—Zn1A—O1A—C9A	2.9 (2)	N2B—Zn1B—O1B—C9B	12.7 (2)
O1D—Zn1A—O1A—C9A	17.1 (7)	N3B—Zn1B—N2B—C11B	−15.95 (19)
N4A—Zn1A—N2A—C17A	−20.83 (19)	O1B—Zn1B—N2B—C11B	108.93 (19)
N3A—Zn1A—N2A—C17A	150.8 (2)	N4B—Zn1B—N2B—C11B	−149.5 (2)
N5A—Zn1A—N2A—C17A	−108.1 (15)	N5B—Zn1B—N2B—C11B	117.2 (13)
O1A—Zn1A—N2A—C17A	−112.5 (2)	N3B—Zn1B—N2B—C10B	−139.1 (2)
O1D—Zn1A—N2A—C17A	69.94 (19)	O1B—Zn1B—N2B—C10B	−14.2 (2)
N4A—Zn1A—N2A—C11A	−141.6 (2)	N4B—Zn1B—N2B—C10B	87.4 (2)
N3A—Zn1A—N2A—C11A	30.02 (19)	N5B—Zn1B—N2B—C10B	−5.9 (14)
N5A—Zn1A—N2A—C11A	131.2 (15)	N3B—Zn1B—N2B—C17B	102.59 (19)
O1A—Zn1A—N2A—C11A	126.8 (2)	O1B—Zn1B—N2B—C17B	−132.5 (2)
O1D—Zn1A—N2A—C11A	−50.80 (19)	N4B—Zn1B—N2B—C17B	−30.95 (19)
N4A—Zn1A—N2A—C10A	99.6 (2)	N5B—Zn1B—N2B—C17B	−124.2 (13)
N3A—Zn1A—N2A—C10A	−88.8 (2)	O1B—Zn1B—N3B—C12B	−68.6 (2)
N5A—Zn1A—N2A—C10A	12.3 (16)	N4B—Zn1B—N3B—C12B	70.3 (2)
O1A—Zn1A—N2A—C10A	7.9 (2)	N5B—Zn1B—N3B—C12B	−174.1 (2)
O1D—Zn1A—N2A—C10A	−169.7 (2)	N2B—Zn1B—N3B—C12B	2.5 (2)
N4A—Zn1A—N3A—C13A	−176.7 (2)	O1B—Zn1B—N3B—C13B	120.1 (2)
N5A—Zn1A—N3A—C13A	−14.4 (3)	N4B—Zn1B—N3B—C13B	−101.0 (2)
O1A—Zn1A—N3A—C13A	82.7 (2)	N5B—Zn1B—N3B—C13B	14.6 (3)
N2A—Zn1A—N3A—C13A	161.6 (2)	N2B—Zn1B—N3B—C13B	−168.8 (2)
O1D—Zn1A—N3A—C13A	−106.1 (2)	N3B—Zn1B—N4B—C19B	131.4 (2)
N4A—Zn1A—N3A—C12A	5.3 (4)	O1B—Zn1B—N4B—C19B	−83.1 (2)
N5A—Zn1A—N3A—C12A	167.6 (2)	N5B—Zn1B—N4B—C19B	14.9 (3)
O1A—Zn1A—N3A—C12A	−95.3 (2)	N2B—Zn1B—N4B—C19B	−160.5 (2)
N2A—Zn1A—N3A—C12A	−16.4 (2)	N3B—Zn1B—N4B—C18B	−53.5 (2)
O1D—Zn1A—N3A—C12A	75.9 (2)	O1B—Zn1B—N4B—C18B	92.0 (2)
N3A—Zn1A—N4A—C19A	173.0 (2)	N5B—Zn1B—N4B—C18B	−170.0 (2)
N5A—Zn1A—N4A—C19A	10.6 (2)	N2B—Zn1B—N4B—C18B	14.6 (2)
O1A—Zn1A—N4A—C19A	−85.5 (2)	N3B—Zn1B—N5B—C23B	−119 (3)
N2A—Zn1A—N4A—C19A	−165.4 (2)	O1B—Zn1B—N5B—C23B	116 (3)
O1D—Zn1A—N4A—C19A	104.3 (2)	N4B—Zn1B—N5B—C23B	16 (3)
N3A—Zn1A—N4A—C18A	−15.9 (4)	N2B—Zn1B—N5B—C23B	108 (3)
N5A—Zn1A—N4A—C18A	−178.4 (2)	C9B—N1B—C1B—C6B	−68.9 (4)
O1A—Zn1A—N4A—C18A	85.6 (2)	C9B—N1B—C1B—C2B	111.4 (3)
N2A—Zn1A—N4A—C18A	5.7 (2)	C6B—C1B—C2B—C3B	0.3 (5)
O1D—Zn1A—N4A—C18A	−84.6 (2)	N1B—C1B—C2B—C3B	180.0 (3)
N4A—Zn1A—N5A—C23A	−40 (3)	C6B—C1B—C2B—C7B	−178.7 (3)
N3A—Zn1A—N5A—C23A	146 (3)	N1B—C1B—C2B—C7B	1.0 (4)
O1A—Zn1A—N5A—C23A	50 (3)	C1B—C2B—C3B—C4B	0.2 (5)
N2A—Zn1A—N5A—C23A	46 (3)	C7B—C2B—C3B—C4B	179.2 (4)
O1D—Zn1A—N5A—C23A	−132 (3)	C2B—C3B—C4B—C5B	−0.9 (6)
C9A—N1A—C1A—C6A	−68.6 (5)	C3B—C4B—C5B—C6B	1.1 (6)
C9A—N1A—C1A—C2A	111.2 (4)	C4B—C5B—C6B—C1B	−0.6 (6)
C6A—C1A—C2A—C3A	0.4 (5)	C4B—C5B—C6B—C8B	179.3 (4)
N1A—C1A—C2A—C3A	−179.4 (3)	C2B—C1B—C6B—C5B	−0.1 (5)
C6A—C1A—C2A—C7A	179.4 (3)	N1B—C1B—C6B—C5B	−179.8 (3)
N1A—C1A—C2A—C7A	−0.4 (4)	C2B—C1B—C6B—C8B	180.0 (3)
C1A—C2A—C3A—C4A	0.1 (5)	N1B—C1B—C6B—C8B	0.3 (5)
C7A—C2A—C3A—C4A	−178.9 (3)	Zn1B—O1B—C9B—N1B	168.3 (2)
C2A—C3A—C4A—C5A	−0.5 (6)	Zn1B—O1B—C9B—C10B	−8.3 (4)
C3A—C4A—C5A—C6A	0.5 (6)	C1B—N1B—C9B—O1B	−7.9 (5)
C2A—C1A—C6A—C5A	−0.4 (5)	C1B—N1B—C9B—C10B	168.9 (3)
N1A—C1A—C6A—C5A	179.4 (3)	C11B—N2B—C10B—C9B	−104.3 (3)
C2A—C1A—C6A—C8A	179.9 (3)	C17B—N2B—C10B—C9B	125.1 (3)
N1A—C1A—C6A—C8A	−0.4 (5)	Zn1B—N2B—C10B—C9B	14.1 (3)
C4A—C5A—C6A—C1A	−0.1 (6)	O1B—C9B—C10B—N2B	−5.3 (5)
C4A—C5A—C6A—C8A	179.7 (4)	N1B—C9B—C10B—N2B	177.9 (3)
Zn1A—O1A—C9A—N1A	170.2 (3)	C10B—N2B—C11B—C12B	144.5 (3)
Zn1A—O1A—C9A—C10A	−14.0 (4)	C17B—N2B—C11B—C12B	−85.1 (3)
C1A—N1A—C9A—O1A	3.1 (5)	Zn1B—N2B—C11B—C12B	25.7 (3)
C1A—N1A—C9A—C10A	−172.8 (3)	C13B—N3B—C12B—C16B	0.3 (5)
C17A—N2A—C10A—C9A	99.8 (3)	Zn1B—N3B—C12B—C16B	−171.4 (3)
C11A—N2A—C10A—C9A	−129.7 (3)	C13B—N3B—C12B—C11B	−176.3 (3)
Zn1A—N2A—C10A—C9A	−16.1 (3)	Zn1B—N3B—C12B—C11B	12.1 (4)
O1A—C9A—C10A—N2A	21.3 (5)	N2B—C11B—C12B—N3B	−26.7 (4)
N1A—C9A—C10A—N2A	−162.7 (3)	N2B—C11B—C12B—C16B	156.8 (3)
C17A—N2A—C11A—C12A	−153.6 (3)	C12B—N3B—C13B—C14B	0.2 (5)
C10A—N2A—C11A—C12A	76.8 (3)	Zn1B—N3B—C13B—C14B	171.3 (2)
Zn1A—N2A—C11A—C12A	−38.7 (3)	N3B—C13B—C14B—C15B	−0.4 (5)
C13A—N3A—C12A—C16A	1.8 (4)	C13B—C14B—C15B—C16B	0.1 (5)
Zn1A—N3A—C12A—C16A	179.9 (2)	N3B—C12B—C16B—C15B	−0.5 (5)
C13A—N3A—C12A—C11A	−179.7 (3)	C11B—C12B—C16B—C15B	175.9 (3)
Zn1A—N3A—C12A—C11A	−1.6 (3)	C14B—C15B—C16B—C12B	0.3 (5)
N2A—C11A—C12A—N3A	29.7 (4)	C11B—N2B—C17B—C18B	156.5 (2)
N2A—C11A—C12A—C16A	−151.8 (3)	C10B—N2B—C17B—C18B	−72.2 (3)
C12A—N3A—C13A—C14A	−0.1 (4)	Zn1B—N2B—C17B—C18B	42.4 (3)
Zn1A—N3A—C13A—C14A	−178.0 (2)	C19B—N4B—C18B—C22B	0.2 (4)
N3A—C13A—C14A—C15A	−1.9 (5)	Zn1B—N4B—C18B—C22B	−175.1 (2)
C13A—C14A—C15A—C16A	2.1 (5)	C19B—N4B—C18B—C17B	−178.2 (3)
C14A—C15A—C16A—C12A	−0.5 (6)	Zn1B—N4B—C18B—C17B	6.5 (3)
N3A—C12A—C16A—C15A	−1.5 (5)	N2B—C17B—C18B—N4B	−36.1 (3)
C11A—C12A—C16A—C15A	−179.9 (3)	N2B—C17B—C18B—C22B	145.5 (3)
C11A—N2A—C17A—C18A	146.0 (3)	C18B—N4B—C19B—C20B	−0.8 (5)
C10A—N2A—C17A—C18A	−84.6 (3)	Zn1B—N4B—C19B—C20B	174.1 (3)
Zn1A—N2A—C17A—C18A	32.1 (3)	N4B—C19B—C20B—C21B	1.2 (5)
C19A—N4A—C18A—C22A	0.4 (4)	C19B—C20B—C21B—C22B	−1.0 (6)
Zn1A—N4A—C18A—C22A	−171.2 (2)	C20B—C21B—C22B—C18B	0.4 (5)
C19A—N4A—C18A—C17A	−177.1 (3)	N4B—C18B—C22B—C21B	0.0 (5)
Zn1A—N4A—C18A—C17A	11.3 (3)	C17B—C18B—C22B—C21B	178.2 (3)
N2A—C17A—C18A—N4A	−30.9 (4)	Zn1B—N5B—C23B—C24B	−129 (51)
N2A—C17A—C18A—C22A	151.6 (3)	O2D—Cl1D—O1D—Zn1A	−38.5 (2)
C18A—N4A—C19A—C20A	0.4 (4)	O4D—Cl1D—O1D—Zn1A	82.4 (2)
Zn1A—N4A—C19A—C20A	171.2 (2)	O3D—Cl1D—O1D—Zn1A	−158.46 (16)
N4A—C19A—C20A—C21A	−0.3 (5)	N4A—Zn1A—O1D—Cl1D	−22.45 (19)
C19A—C20A—C21A—C22A	−0.4 (5)	N3A—Zn1A—O1D—Cl1D	179.11 (19)
C20A—C21A—C22A—C18A	1.2 (5)	N5A—Zn1A—O1D—Cl1D	77.49 (19)
N4A—C18A—C22A—C21A	−1.2 (5)	O1A—Zn1A—O1D—Cl1D	−116.4 (5)
C17A—C18A—C22A—C21A	176.2 (3)	N2A—Zn1A—O1D—Cl1D	−102.37 (19)
Zn1A—N5A—C23A—C24A	−46 (40)		

### Hydrogen-bond geometry (Å, º)

**Table:**

*D*—H···*A*	*D*—H	H···*A*	*D*···*A*	*D*—H···*A*
N1*A*—H1*A*···O3*D*^i^	0.88	2.00	2.868 (3)	171
N1*B*—H1*B*···O4*E*^ii^	0.88	2.15	2.979 (3)	157
N1*B*—H1*B*···O3*E*^ii^	0.88	2.41	3.138 (4)	141

Symmetry codes: (i) *x*, −*y*+1, *z*+1/2; (ii) *x*, −*y*, *z*+1/2.

## References

[bb1] Allen, F. H. (2002). *Acta Cryst.* B**58**, 380–388. 10.1107/s0108768102003890 12037359

[bb2] Bruker (2007). *APEX2*, *SAINT-Plus* and *SADABS* Bruker AXS Inc., Madison, Wisconsin, USA.

[bb3] Macrae, C. F., Bruno, I. J., Chisholm, J. A., Edgington, P. R., McCabe, P., Pidcock, E., Rodriguez-Monge, L., Taylor, R., van de Streek, J. & Wood, P. A. (2008). *J. Appl. Cryst.* **41**, 466–470.

[bb4] Makhov, P., Golovine, K., Uzzo, R. G., Rothman, J., Crispen, P. L., Shaw, T., Scoll, B. J. & Kolenko, V. M. (2008). *Cell Death Differ.* **15**, 1745–1751. 10.1038/cdd.2008.106 PMC2585550 18617897

[bb5] Marlin, D. S., Cabrera, D. G., Leigh, D. A. & Slawin, A. M. Z. (2006). *Angew. Chem. Int. Ed.* **45**, 77–83.

[bb6] Patten, T. E., Olmstead, M. M. & Troeltzsch, C. (2008). *Inorg. Chim. Acta*, **361**, 365–372.

[bb7] Sheldrick, G. M. (2008). *Acta Cryst.* A**64**, 112–122. 10.1107/S0108767307043930 18156677

[bb8] Spek, A. L. (2009). *Acta Cryst.* D**65**, 148–155. 10.1107/S090744490804362X PMC2631630 19171970

[bb9] Xu, Z., Baek, K.-H., Kim, H. N., Cui, J., Qian, X., Spring, D. R., Shin, I. & Yoon, J. (2010*a*). *J. Am. Chem. Soc.* **132**, 601–610. 10.1021/ja907334j 20000765

[bb10] Xu, Z., Han, S. J., Lee, C., Yoon, J. & Spring, D. R. (2010*b*). *Chem. Commun.* **46**, 1679–1682. 10.1039/b924503k 20177614

